# Noncontact assessment for fatigue based on heart rate variability using IR-UWB radar

**DOI:** 10.1038/s41598-022-18498-w

**Published:** 2022-08-20

**Authors:** Sarfaraz Ahmed, Yonggu Lee, Young-Hyo Lim, Seok-Hyun Cho, Hyun-Kyung Park, Sung Ho Cho

**Affiliations:** 1 Department of Electronics and Computer Engineering, College of Engineering, Hanyang University, 04763 Seoul, Republic of Korea; 2 Division of Cardiology, Department of Internal Medicine, College of Medicine, Hanyang University, Seoul, 04763 Republic of Korea; 3 Department of Otorhinolaryngology, College of Medicine, Hanyang University, Seoul, 04763 Republic of Korea; 4 Department of Pediatrics, College of Medicine, Hanyang University, Seoul, 04763 Republic of Korea

**Keywords:** Health care, Engineering

## Abstract

Physical fatigue can be assessed using heart rate variability (HRV). We measured HRV at rest and in a fatigued state using impulse-radio ultra wideband (IR-UWB) radar in a noncontact fashion and compared the measurements with those obtained using electrocardiography (ECG) to assess the reliability and validity of the radar measurements. HRV was measured in 15 subjects using radar and ECG simultaneously before (rest for 10 min before exercise) and after a 20-min exercise session (fatigue level 1 for 0–9 min; fatigue level 2 for 10–19 min; recovery for ≥ 20 min after exercise). HRV was analysed in the frequency domain, including the low-frequency component (LF), high-frequency component (HF) and LF/HF ratio. The LF/HF ratio measured using radar highly agreed with that measured using ECG during rest (ICC = 0.807), fatigue-1 (ICC = 0.712), fatigue-2 (ICC = 0.741) and recovery (ICC = 0.764) in analyses using intraclass correlation coefficients (ICCs). The change pattern in the LH/HF ratios during the experiment was similar between radar and ECG. The subject’s body fat percentage was linearly associated with the time to recovery from physical fatigue (R^2^ = 0.96, *p* < 0.001). Our results demonstrated that fatigue and rest states can be distinguished accurately based on HRV measurements using IR-UWB radar in a noncontact fashion.

## Introduction

Autonomic nervous system (ANS) activity can be indexed using heart rate variability (HRV). Clinical application of HRV has been broadened in predicting the prognosis of cardiovascular diseases and the development of metabolic illness^1,2^. Recent research suggested that HRV could be a useful marker of fatigue resulting from physical exercise and could therefore be utilized to optimize physical training loads and improve performance in sports. HRV is typically measured using electrocardiography (ECG), which requires a form of contact sensor to measure electrical potentials on the body surface. Needs for reliable noncontact vital sign monitors have emerged because of local troubles on the skin, immobilization and discomfort of patients and the spread of contagious diseases within health care facilities caused by the contact electrodes and wires used for ECG^3^. The recent outbreak of COVID-19 pandemic has even more urged to develop and improve non-contact vital signs monitors, as the social distancing was recognized as an important strategy to contain the infection^4^. In the field of sports medicine, noncontact assessment tools for physical fatigue are also desired, given that any contact sensors may impede athletes’ performance in competitive sports. However, to date, only a few studies have been reported on noncontact methods for HRV measurements, and fewer have been reported on the application of noncontact HRV measurements to assess fatigue levels^5,6^.

Previous studies reported that metabolic characteristics, including obesity, insulin resistance and high blood pressure, were associated with the ANS activity status, which is reflected in HRV. Body composition, including total body fat (BF) mass, has also been reported to correlate with HRV parameters^7^. Because body composition is an important factor that determines athletic performance in many sports and HRV measurements can reflect both the fatigue level and BF mass, we hypothesized that the association between BF mass and the ability to recover from physical fatigue could be reflected in the changing pattern of HRV measurements.

Therefore, in this study, we measured HRV during rest and fatigued states before and after physical exercise using an IR-UWB radar sensor, investigated the accuracy of the measurements by comparing them with HRV measured using an ECG sensor, and evaluated the feasibility of radar-measured HRV as a marker of fatigue caused by physical exercise. Additionally, it is easier to record and store HRV data obtained using radar compared to ECG. We can use these recorded exercise data for fatigue prediction in future analyses of daily exercise habits, which is more convenient than using ECG. We also investigated the relationship between BF percentage and the ability to recover from physical fatigue by measuring the change patterns of HRV obtained using radar in a noncontact fashion.

## Methods

### Subjects

Fifteen healthy volunteers participated in the study. All participants were advised to cease alcohol intake and smoking and to avoid any strenuous physical activities for at least 24 h before the experiment. They were also advised to have 4 h of fasting before the experiment. Body compositions were measured using a bioimpedance method, and ECG was obtained before IR-UWB radar measurement in all participants. Participants with heart rhythms other than sinus rhythm were excluded from the study to ensure appropriate measurements of HRV. Informed consent was obtained from all participants before they entered the study. The study protocols were reviewed and approved, and the study procedures were monitored by the Institutional Review Board of Hanyang University (IRB No. 2021-01-015). All experimental procedures were performed in accordance with relevant guidelines and regulations.

### Experimental setting

**Figure 1:**
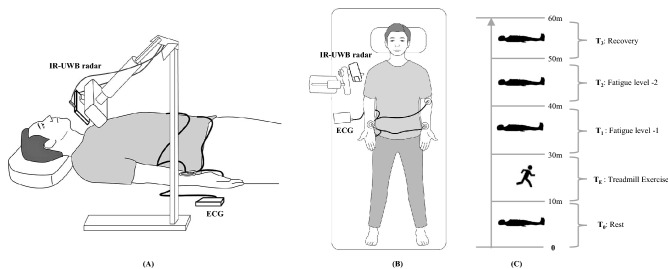
Illustration of HRV measurement using IR-UWB radar. (**A**) Side view: The measurement setting. The radar transmitter was positioned at a 50-cm distance from the point 2 cm below the right mandibular angle and at a 45-degree angle with the bed on all three planes (coronal, sagittal. transverse). The HRV were measured in a supine position before and after the treadmill exercise using ECG and IR-UWB radar sensor simultaneously and data was captured from the carotid artery area. (**B**) Top view: with same setting of ECG and radar. (**C**) Experimental phases. The measurement was obtained in a supine position for 10 min before the exercise, then subjects underwent a 20-min’s treadmill exercise, and the measurement resumed in the same supine position for 30 min (the first 10 min for fatigue level 1, the second 10 min for fatigue level 2 and the third 10 min for recovery). Total time of experiment was of 1-h. This figure has been drawn and modified on software “Adobe Illustrator CC”.

The experimental setting is depicted in Fig. 1. Each participant underwent 1-h experimental protocols, including a 10-min preexercise measurement, a 20-min treadmill exercise session and a 30-min post-exercise measurement. The heartbeat signal from the carotid artery was measured in a supine position before and after treadmill exercise using IR-UWB radar and ECG simultaneously. The measurements started 1 min after a participant assumed a supine position, when the participant’s heart rate was stabilized. First, HRV was measured in a supine position for 10 min before exercise (rest; T_0_). Then, a participant underwent treadmill exercise at 8 km/h speed for 20 min to simulate a fatigue state caused by physical exertion (exercise; T_E_). After finishing the exercise, the participant was placed in the supine position again and rested for 1 min until body movements were minimized and respiration and heart rate were stabilized. Then, HRV measurement was resumed and continued for 30 min. The measurement period after exercise was divided into 3 phases according to the elapsed time as follows: fatigue level 1 (1–10 min postexercise, T_1_), fatigue level 2 (11–20 min postexercise T_2_) and recovery (21–30 min postexercise, T_3_). Participants were advised to minimize their body movements and breathe naturally throughout the measurements. For data processing, 2 min of data (1 min at the start and 1 min at the end) were excluded from each phase to avoid unnecessary noises and artefacts, and 8-min measurement data were used in the analyses of HRV.

For the experiment, an industrial-based IR-UWB radar transceiver (XK300-VSA, Xandar Kardian, Toronto, Canada) was used to measure heartbeat. The radar sensor has a centre frequency of 8.7 GHz, a bandwidth of 1.5 GHz, an ADC pulse repeating frequency of 23.3 GHz and a peak pulse output power of 6.3 dBm. The sampling rate for both the ECG sensor and IR-UWB radar sensor was set to 250 samples/second.

The radar sensor was placed at a 50-cm distance from the surface of the right neck and at a diagonal angle of 45 degrees with all 3 body planes (coronal, sagittal. transverse) and aimed at the carotid bulb as previously described. This position and aiming angle allow the radar sensor to detect carotid artery pulsation without being interfered with by respiratory signals, which is typically overwhelming, when measured using the radar sensor on the anterior chest surface under normal breathing conditions. The radar sensor can detect heartbeat from either the neck or the anterior chest in various body positions including sitting, standing, supine and prone positions but this position provided most accurate estimates for the R-R intervals^8^.

### ECG sensor measurement

A single bipolar lead device (PSL-iECG2, Physio Lab, Pusan, Republic of Korea) was used to record ECG. Three attachable electrodes were placed on the subject’s left upper arm, left wrist and right wrist. The R-R interval data recorded using the ECG device were also processed and analysed in MATLAB to produce standard HRV values.

### IR-UWB radar data processing

**Figure 2:**
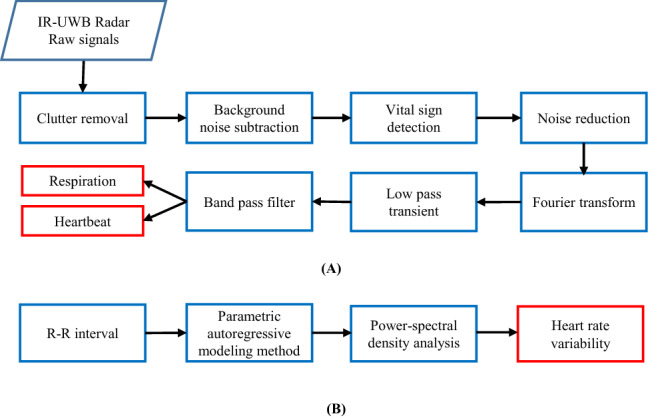
Digital signal processing algorithm for the HRV extraction using IR-UWB radar. (**A**) In the first phase, clutter and background subtraction were removed to make raw signal more appropriate for an accurate vital sign measurement. Next, fast Fourier transform (FFT) and a band pass filter was applied to raw data to obtain respiration and heartbeat signal. (**B**) In the second phase, Heart rate variability (HRV) was measured from the heartbeat rate by detecting R-R interval. A parametric method was applied on the R-R interval to estimate power spectral density (PSD) and find HRV components including LF and HF. The LF/HF ratio was calculated to index the states of rest, fatigue and recovery.

The algorithms used to process the data acquired from the radar sensor and to extract HRV from the data are summarized in Fig. 2. All data processing was conducted using the commercial software MATLAB (Math-works, Natick, Massachusetts, USA).

The IR-UWB radar device was equipped with a single transmitter emitting pulses of radio waves with a narrow band towards the target and a single receiver collecting signals reflected from the target. The received signals consisted of raw data filled with unwanted echoes from the surroundings called clutters. We removed these clutters using a loopback filter. The direct current (DC) value was used to remove the remaining noise. The received signal after clutter removal can be described with the following equations:

1 $${\varvec{c}}_{{\varvec{i}}} \left( {\varvec{t}} \right) = \user2{\alpha c}_{{{\varvec{i}} - 1}} \left( {\varvec{t}} \right) + \left( {1 - {\varvec{\alpha}}} \right){\varvec{r}}_{{\varvec{i}}} \left( {\varvec{t}} \right),$$

2 $${\varvec{y}}_{{\varvec{i}}} \left( {\varvec{t}} \right) = {\varvec{r}}_{{\varvec{i}}} \left( {\varvec{t}} \right) - {\varvec{c}}_{{\varvec{i}}} \left( {\varvec{t}} \right),$$

where ${{\varvec{c}}}_{{\varvec{i}}}\left({\varvec{t}}\right)$ is the clutter signal, *α* is the estimated ratio of the received signal to the clutter signal, ${{\varvec{r}}}_{{\varvec{i}}}\left({\varvec{t}}\right)$ is the received signal that contains the information about the target, and *i* and *t* are the slow and fast time indices, respectively.

**Figure 3:**
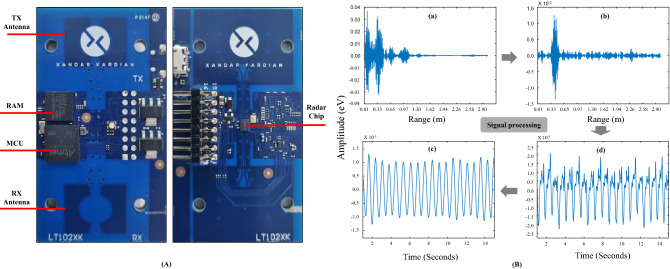
Detecting system and data flow for heartrate measurement using IR-UWB radar sensor. (**A**) IR-UWB radar detecting system (front and back). In the front side, TX/RX Antenna, RAM memory and Microcontroller unit (MCU) are embedded; while in the back side radar chip are embedded in the detecting system. (**B**) Data stream from the IR-UWB radar sensor. (**a**) A raw radar signal was extracted after locating the target in the range with respect to time. (**b**) The raw signal, comprises of both respiratory and heartbeat signal. (**b**) A moving target indication (MTI) filter was used to remove clutter and background noise from the captured raw data. (**c**) A vital signs signal was acquired in the target range along the slow time axis by rasping the data. (**d**) The extracted vital signals were sent to the respective filters and processed using the fast Fourier transformation (FFT) to produce RR and HR. Finally, an accurate HR signal was obtained for further measuring HRV indexes.

Changes in typical heartbeat signals obtained from the radar sensor during the data processing and detecting system are depicted in Fig. 3. The actual raw data was in the form of 2D time-range signal. During data flow we picked up certain distance which we believe shows best observation of carotid pulses, on that distance we collected radar signal. This radar signal was basically 1D (range) signal with respect to time. Raw signals obtained from the target containing carotid pulse and respiration had environmental noise and some artefacts, but after clutter removal and background subtraction, the reflected signals from the carotid artery were recognizable; subsequently, the relative movement index was calculated and target location was identified. After ranging the target, the vital signal was obtained from the radar signal, including the respiratory and carotid pulse signals. Normally HR carries high frequency ranges (0.8 ~ 2.3 Hz) and RR carries (0.08 ~ 1.0 Hz). The heartbeat signal contains harmonic components in the frequency domain. By FFT we found those harmonic components and converted them in the pulses. The signals for RR and HR were passed through their respective filters, and fast Fourier transform (FFT) was simultaneously applied to the signals, providing estimates of RR and HR.

After the carotid pulse was successfully extracted, the beat-to-beat (R-R) interval was measured to compute HRV in frequency domains, including the very low-frequency component (VLF), low-frequency components (LF), high-frequency components (HF) and the LF/HF ratio. The P-Welch parametric method was used to compute the power spectral density components of HRV. Finally, data analyses were performed to determine whether these HRV values measured using radar could distinguish fatigue from rest. HRV frequencies in the ranges of 0–0.04 Hz, 0.04–0.15 Hz and 0.15–0.40 Hz were defined as the VLF, LF, and HF ranges, respectively.

### Measurement for percent body fat

The BF percentage was measured using a bioimpedance method (Biospace Co., Ltd, Seoul, Korea) in all subjects before any HRV was measured using radar and ECG. Before the bioimpedance measurement, all subjects were advised to avoid engaging in strenuous exercise and drinking alcohol for at least 12 h. The bioimpedance measurement was obtained for 2 min while subjects were standing on both bare feet on the electrode panel, gripping the left and right electrode handle with the respective hands and abducting both arms 30 degrees away from the torso.

### Statistical analysis

Subject characteristics, including age, weight, height, BMI and body composition, are described as the mean ± SD. The agreement levels between HRV parameters measured using radar and those measured using ECG in each phase were tested using intraclass correlation coefficients (ICCs) and Bland–Altman (BA) plots with mean bias and 95% limit of agreement (LOA) values. An ICC < 0.75 was considered modest, an ICC of 0.75–0.89 was considered good, and an ICC ≥ 0.90 was considered excellent in representing reliability between the two methods. Repeated measures ANOVA was performed to identify the influence of the continuously changing HRV values during the experimental phases using both ECG and radar HRV measurement methods.

To understand the relationship between the BF percentage and patterns of recovery from exercise-induced fatigue, elapsed times to the recovery state were estimated. We defined the time to recovery as the time for the LF/HF ratio to return to a level below the upper limit of the 95% confidence interval (CI) of the LF/HF ratios measured using radar at rest. We calculated the LF/HF ratio every minute after the end of exercise using a 3-min moving window for power spectral density (PSD) analyses. The LF/HF ratio obtained from HRV data between *t *− 1 and *t* + 1 min after the end of exercise was considered the LF/HF ratio at *t* minutes after exercise. Then, we plotted the LF/HF ratios against time after the end of exercise and estimated the time to recovery using an exponential decay curve fit as follows:

3 $${\varvec{E}}\left( {\varvec{x}} \right) = {\varvec{a}}*{\mathbf{exp}}\left( {{\varvec{b}}*{\varvec{x}}} \right)$$

where *a* and *b* are the coefficients with 95% confidence bounds.

Finally, a linear regression model was applied to identify the relationship between the time to recovery from exercise-induced fatigue and the BF percentage.

All statistical analyses were performed using R-4.1.2 (R Core Team, R Foundation for Statistical Computing, Vienna, Austria), RStudio-1.3 (RStudio Team, Rstudio, BPC, Boston, MA, US) and MATLAB-R2021b (Math-works, Natick, Massachusetts, USA). A *p value* < 0.05 was considered statistically significant. Stata MP 17 (Stata Corp LLC, Texas, US) statistical software was used for the statistical power analysis to demonstrate the feasibility of radar. PASS 2008 (NCSS, LLC, Kaysville, US) sample size software was used to estimating sample size or determining the power of a statistical test. All the statistical analysis was performed over 15 number of subjects.

## Results

### HRV analysis in the frequency domain

Subject characteristics and HRV indices are properly defined in Table 1. The HRV indices, LF, HF and LF/HF ratio for each phase were estimated by PSD measurement. The age of the subjects was 27.2 ± 3.7 years, including 80% of them were male. The mean BMI was 23.54 ± 2.6 kg/m^2^, the total BF mass was 18.3 ± 3.9 kg, and the BF percentage was 24.6 ± 5.1%. For the PSD of HRV measured using ECG and radar, the LF, HF and LF/HF ratio for T0, T1, T2 and T3 are shown in Table 1. The pattern of the decaying LF/HF ratio from T1 to T3 can be seen from both the ECG and radar methods. In the final T3 phase, the HRV indices were close to those in the T0 phase, which corresponded to fatigue recovery. Low values of standard mean deviation (SMD) indicate that the HRV indices were clustered closely around the mean (more reliable).

**Table 1:** Subject characteristics.

Clinical characteristics		Values (N = 15)	
Age (years)		27.2 ± 3.7	
Gender (male/female)		13/2	
Weight (kg)		70.9 ± 10.8	
Height (cm)		172.7 ± 6.5	
BMI (kg/m^2^)		23.54 ± 2.6	
Total body fat mass (kg)		18.3 ± 3.9	
Percent body fat (%)		24.6 ± 5.1	

HRV variable	ECG	Radar	SMD
**T0: Rest**			
LF (second^2^/Hz)	12.9 ± 4.2	10.1 ± 3.5	0.771
HF (second^2^/Hz)	11.5 ± 4.0	8.62 ± 4.0	0.739
LF/HF	1.20 ± 0.3	1.25 ± 0.4	− 0.189
**T1: Fatigue 1**			
LF (second^2^/Hz)	8.61 ± 6.5	8.67 ± 7.10	− 0.089
HF (second^2^/Hz)	2.68 ± 2.2	2.60 ± 2.35	0.030
LF/HF	3.43 ± 0.7	3.67 ± 0.95	− 0.293
**T2: Fatigue 2**			
LF (second^2^/Hz)	4.56 ± 1.2	5.1 ± 2.4	− 0.312
HF (second^2^/Hz)	2.40 ± 0.5	2.4 ± 0.8	− 0.020
LF/HF	1.95 ± 0.4	2.1 ± 0.5	− 0.355
**T3: Figure 3**			
LF (second^2^/Hz)	11.7 ± 4.8	9.7 ± 5.5	0.383
HF (second^2^/Hz)	7.70 ± 4.1	6.8 ± 4.58	0.217
LF/HF	1.70 ± 0.5	1.6 ± 0.40	0.184

Data were shown as the mean ± SD or N (%).

**Figure 4:**
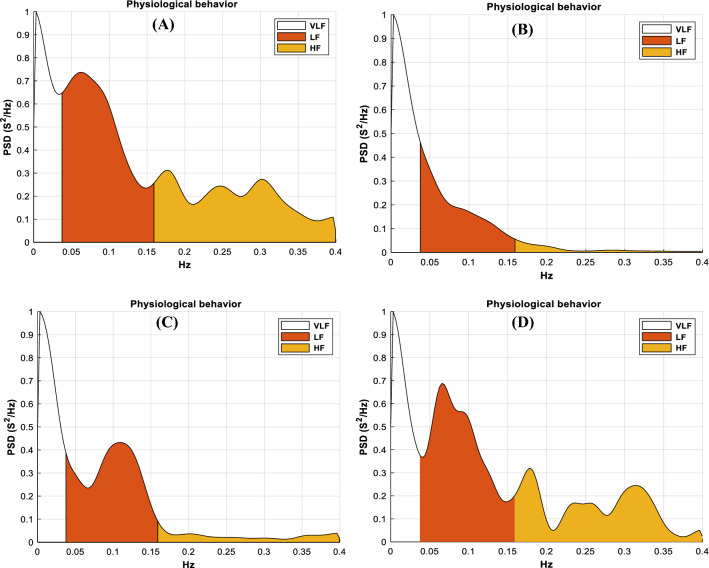
Power spectrum distribution of HRV using IR-UWB radar. (**A**) HRV distribution in LF and HF region when subject was on supine position before exercise (Rest). (**B**) HRV distribution in LF and HF region when subject was on supine position during 0 ~ 10 min of post-exercise (Fatigue-1). (**C**) HRV distribution in LF and HF region when subject was on supine position during 10 ~ 20 min of post-exercise (Fatigue-2). (**D**) HRV distribution in LF and HF region when subject was on supine position during 20 ~ 30 min of post-exercise (Recovery).

A typical PSD pattern of HRV measured in a subject using IR-UWB radar is depicted in Fig. 4. At T0, the area in the HF range was relatively large (Fig. 4A). Early after the end of exercise (T1), the PSD shifted to the VLF range, and the peaks within the HF range disappeared while the small LF range remained (Fig. 4B). The area in the LF range decreased slightly, whereas the area of the HF range markedly decreased; thus, the LF/HF ratio increased. As time passed (T2), the area of the LF range increased, and the peak in the LF range nearly returned (Fig. 4C). After 20 min passed (T3), the area in both the LF range and the HF range increased substantially, and the PSD pattern resembled the PSD pattern observed at rest (Fig. 4D).

To evaluate the agreement between radar and ECG, ICC and BA plots were used (Table 2, Supplementary Fig. 1). ICCs showed that the LF/HF ratios measured using radar had, in general, a good level of reliability compared to those measured using ECG in all phases (Table 2). The ICCs between radar and ECG ranged between 0.70 and 0.85 and were highest at T0 and lowest at T1. The BA plots showed high levels of agreement between the LF/HF ratios measured using radar and those measured using ECG in all phases. BA plots showed that the mean biases between radar and ECG were not significant in all phases. The widths of 95% LOAs were narrowest at T0 (0.79), then increased to the widest level in T1 phase (3.36), and gradually decreased close to that at T0 through T2 (1.33) and T3 phase (1.18) (Supplementary Fig. 1). There was no proportional bias on the distribution of data around the mean difference line in any phase.

**Table 2:** Agreement between HRVs from radar and those from ECG.

Phase	Linear model	Correlation	ICC			Bland–Altman plot		
	*β/*C*	Pearson’s R (*p* value)	ICCR	95% CI	Mean bias	*p* value for mean bias	Upper 95% LOA	Lower 95% LOA
Rest (T0)	0.91/0.16	0.816 (0.002)	0.807	0.527–0.930	0.062 (− 0.060–0.188)	0.249	0.46	− 0.33
Fatigue 1 (T1)	− 0.18/1.12	0.779 (0.006)	0.712	0.345–0.892	0.232 (− 0.142–0.607)	0.159	1.41	− 1.95
Fatigue 2 (T2)	0.52/0.82	0.781 (0.005)	0.741	0.399–0.904	0.177 (− 0.034–0.388)	0.064	0.84	− 0.49
Recovery (T3)	0.55/0.62	0.783 (0.004)	0.764	0.442–0.913	− 0.078 (− 0.267–0.110)	0.336	0.51	− 0.67

*Beta coefficient and intercept of the linear models.

*ICC* Intraclass correlation coefficient, *LOA* Limit of agreement, *HRV* Heart rate variability, *ECG* Electrocardiography.

**Figure 5:**
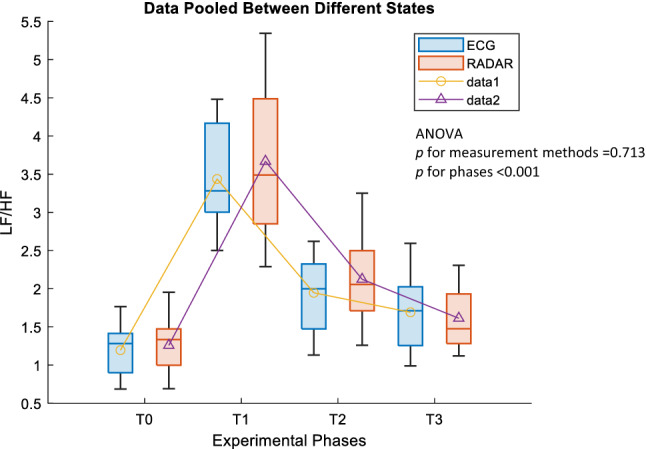
Comparison of LF/HF ratios between ECG and radar.The changing pattern of LF/HF ratios during the experiment was not significantly different between measurement methods whereas it significantly increased in T1 and T2 and returned to the level similar to T0 in T3. The line plots indicate the mean values and boxplots indicate the median and interquartile rage. T0 for Rest (pre-exercise); T1 for Fatigue-1 (post-exercise); T2 for Fatigue-2 (post-exercise); T3 for Recovery (post-exercise).

The mean values of the LF/HF ratios measured using both radar and ECG were close to 1.5 at T0, then markedly increased more than twofold in T1 and gradually decreased as time passed. In recovery phase T3, the mean values of LF/HF ratios reached approximately 1.5 in both measurement methods (Fig. 5). There was no significant difference in the change pattern of the LF/HF ratios between radar and ECG (*p value* in ANOVA = 0.713).

**Figure 6:**
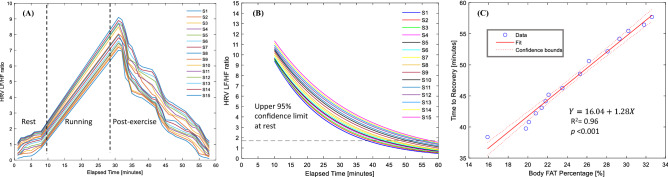
Decay rates of the increased LF/HF ratios after the end of exercise and total body fat mass. (**A**) Changing patterns of the LF/HF ratios. LF/HF ratios measured every 1 min markedly increased after exercise and gradually decreased since the end of exercise, which resembles an exponential decrease pattern. (**B**) The estimates of the LF/HF ratio. The estimates of the LF/HF ratios were derived from an exponential decay model. Time to recovery was estimated at the point where a decaying curve crosses the upper 95% confidence limit (the broken line) of the LF/HF ratios at rest. (**C**) Relationship between body fat percentage and the time to recovery. The body fat percentage is linearly associated with the time to recovery.

After the end of exercise, the LF/HF ratios measured using radar decreased gradually over time and eventually reached the upper limit of the 95% CI of the LF/HF ratio at rest in all subjects (Fig. 6). We used an exponential decay model to fit the data and to identify the time to recovery within each subject. Exponential curve fitting was used because we had linearly and nonlinearly distributed values in the original datasets (Fig. 6A); therefore, for the regression model, estimated values were used (Fig. 6B). A linear regression model showed that the time to recovery within individual subjects estimated using the exponential decay models was tightly associated with the BF percentage (Fig. 6C).

Because some analyses could have been underpowered due to the small subject number, we calculated statistical powers of the repeated measures ANOVA and ICCs using the statistical software, Power Analysis and Sample Size (PASS) 2008. The repeat measures ANOVA was sufficiently powered (> 0.9), while ICCs were underpowered for a moderate reliability margin of 0.5 (approximately 0.3–0.7).

## Discussion

The purpose of this study was to investigate whether a contactless IR-UWB radar sensor is feasible for identifying physical fatigue due to exercise and the association between body composition and the time to recovery from fatigue through HRV measured using radar. In our study, we found that (1) the LF/HF ratio of HRV measured using IR-UWB radar was highly reliable, accurate and convenient compared to that measured using ECG; (2) the typical increase–decrease pattern of changes in the LF/HF ratios after 20 min of medium-intensity exercise could be reliably identified using radar; and (3) the subject’s BF percentage was highly associated with the time to recovery from the physical fatigue estimated with the LF/HF ratios measured using radar.

This study can tell whether there is potential usage for radar to be a fatigue monitor because we know from the results that fatigue can be represented by the typical increase in the LF/HF ratio. The measurements of LF/HF ratios using radar showed fairly close results compared with the measurements using ECG in all phases T0, T1, T2 and T3 (Table 1). The SMDs showed some differences in the mean values between ECG and radar; however, all absolute SMD values were > 0.1. Among all variables, the SMD of the LF/HF ratio was the value of most interest. The LF/HF ratio increased in fatigues states and then decreased again in recovery. In the frequency domain analysis (Fig. 5), there was a significant increase in the LF/HF ratio after exercise. This could be attributed to physical fatigue, which reduces vagal activity and enhances sympathetic activity as part of arterial pressure control mechanisms after exercise^9^. As time passes after exercise, the LF/HF ratio gradually decreases to a level similar to that at rest, which reflects reactivation of parasympathetic activity and withdrawal of sympathetic activity. This increasing and decreasing pattern of changes in the LF/HF ratio was observed to be very similar between the two methods, representing the potential utility of radar as an effective fatigue monitor. The changes occurring in HRV according to exercise intensity (moderate and vigorous) using ECG were examined in^10^; however, in this study, they focused on ultralow frequency and very-low frequency components of HRV and did not include recovery assessment after treadmill running.

In our findings, the percentage mean biases using both ECG and radar (3.1 and 3.3%, respectively) and ICCs (0.807 and 0.764, respectively) in the T0 and T3 phases were remarkably better than those in the T1 and T2 phases (4.6 and 5.2%, respectively) and ICC (0.712 and 0.741, respectively). This implies that the level of agreement between ECG and radar was relatively higher in the T0 and T3 phases than in the fatigue T1 and T2 phases (Table 2, Supplementary Fig. 1). Heartbeat signal right after exercise comprise of some artefacts and unwanted noise, such fluctuation in the heartbeat signal finally causes increased in the mean biases of T1 and T2 states. It could be because of unstable heartbeat signal right after exercise which eventually get stable in some time. These abnormalities were challenged to the algorithm to diminish completely; however, most of them were eliminated by applying specialized filtering and techniques while detecting heart rates^11^. The mean biases in the T1 and T2 states was higher than T0 and T3 states, because T0 and T3 states have more stable heartbeat rate. Although the level of mean biases increases in the T1 and T2 states, the increased levels of the bias will not affect the feasibility of radar as a fatigue monitor because the LF/HF ratio in T1 and T2 is markedly different (close to threefold and twofold, respectively) from that in T0. In contrast, small abnormalities were easily removed in low heart rates, which tends to lower HRV in the rest and recovery phases (T0, T3). Such consideration depicts the proportional bias of the T0 and T3 phases in addition to the relatively lower LOA in the T1 and T2 phases. Moreover, a tight association was found between BF percentage and recovery time in the linear regression model. An exponential decay trend was predicted based on the real dataset, which represents the decay of the LF/HF ratio from fatigue to recovery at every minute interval (Fig. 6). The idea of an exponential decay pattern of the LF/HF ratio was taken from the postexercise cardiac ANS recovery data explained in^12^. The steady decline in heart rate from exercise to recovery was also deliberated in^13,14^. After the estimation of exponential decay of the LF/HF ratio, a regression analysis was performed, and it was observed that subjects who had a lower BF percentage recovered faster than those who had a higher BF percentage. Although this is not always true, as there might be an additional BMI parameter that could impact HRV recovery, in our research, we mainly focused on BF percentage, which is a key factor in fatigue recovery. All the above results suggest that treadmill running can acutely affect HRV and that BF percentage plays an important role in fatigue recovery.

To date, only a few studies have reported on the measurement of HRV using radar technology. Heartbeat was measured without contact with the skin using capacitive coupled devices placed in the bed^15^; however, in this method, the device and skin must be very close, unlike radar, which can measure heartbeat signals at a distance of 0.5 m. In^16–18^, HRV was assessed by placing a radar in the chest wall, but high noise from the respiration signal decreased the sensitivity of radar for HRV measurement. Basically, the accuracy of HRV measurements depends on the accuracy of R-R interval measurements. To obtain accurate LF/HF rates, beat-to-beat interval measurements should be highly precise. We introduced an accurate method to measure the R-R interval from carotid artery pulsation using IR-UWB radar in a previous study^19^. The current study is the first to extend the radar measurement method to obtain HRV parameters to assess physical fatigue levels after exercise. A signal taken from the carotid artery contains less of an effect of respiration noise, which may help in the accurate measurement of the LF/HF ratio. Measurement of HRV parameters using sensors requiring physical contact, including ECG and photoplethysmography, has been standard for assessing fatigue levels^20^, but it can cause discomfort to subjects engaging in strenuous activities and thus is impractical. In contrast, our results showed that IR-UWB radar was feasible for obtaining accurate measurements of HRV parameters from those subjects in a noncontact fashion.

In this study, we also assessed the feasibility of HRV measurements using radar as a predictor of body composition, which may affect the performance of subjects engaging in exercise. We observed and analysed the impact of the body composition parameter (BF percentage) on fatigue recovery. The relationship of HRV with body composition parameter (BMI) was considered in^21^; however, in this study, weight was a key factor instead of BF percentage, and they did not investigate any regression of recovery after exercise. Body composition, including obesity, is also an HRV marker for children with disturbances in body weight^22^; however, how HRV recovery is associated with body weight has not been examined. An effect of stretching exercise on HRV was found in^23^, and recovery was also discussed; however, such study may be good for low-level exercises effect on HRV. ANS activity recognition, such as stress, was measured using radar by referring to HRV components^24,25^; therefore, further activities such as rest, fatigue, drowsiness and emotion classification using novel radar technology are still under debate. In our study, we assessed whether radar can be a fatigue predictor in relation to body composition parameter (BF percentage) and LF/HF ratios. By using regression and correlation findings, a device can recommend either stopping or resuming exercise based upon fatigue recovery time. The device can categorize the subjects into groups with respect to their body fat mass and recovery time such that a target for body fat mass reduction can be given to a subject so that the subject could be employed in the optimal group.

Here, we focus on the application and future perspective of fatigue measurement using radar technology. Apparently, noncontact devices are very useful in biomedical and health care applications. Physically connecting the ECG leads to check the health condition of people in a gym or during workout is uncomfortable, although their condition can be assessed without wearing any physical device using IR-UWB radar. IR-UWB radar may be used for early warning of drowsiness during car driving, which is required to diminish tiredness-linked driving accident rates. This research work may extend to investigate the proper amount of physical exercise for an individual by classifying fatigue into multiple exercise levels. However, we did not provide any diagnostic criteria for fatigue state. We can do this in our future work by measuring HRV at multiple levels of exercise strength in subjects engaging in exercise.

## Limitations

First, our study investigated the feasibility of radar as a physical fatigue assessment tool through its ability to measure HRV accurately; however, our experiments did not provide any standards or criteria for the diagnosis of a fatigue state from a certain physical activity. Further experiments with exercise protocols of various types and intensities are required to develop criteria for the fatigue state. Second, the typical increase–decrease in the LF/HF ratio pattern that we observed after exercise resulted from 15 volunteers, including 2 females. The decay pattern of the LF/HF ratio peaks may be different in study populations with different characteristics from ours, particularly in athletes who regularly exercise. Moreover, the number of subjects was relatively small, therefore, the assessment for ICCs was not sufficiently powered. To broaden applications of radar in assessing fatigue states, further studies are required on larger number of and more various subsets of the population. Third, we measured HRV in a supine position using a radar sensor aiming at the neck at a certain angle and distance. To develop radar as a fatigue assessment tool for athletes, these stiff requirements for radar measurement should be improved, and the feasibility of radar should be evaluated in other postures.

## Conclusion

IR-UWB radar can accurately measure HRV parameters based on carotid artery pulsation in a noncontact fashion. By providing accurate HRV measurements and typical patterns of change in the LF/HF ratios, IR-UWB radar may be useful to assess fatigue and recovery states induced after physical exercise. Our results investigated the feasibility of IR-UWB radar as a marker for physical fatigue following a certain level of exercise. Secondly, BF percentage is highly associated with the time to recovery from physical exercise estimated through HRV measured using radar. Our results indicate the potential roles of radar in assessing physical fatigue and recovery from fatigue for individualized exercise programs and the possibility of assessing body compositions using radar in a noncontact fashion.

## Supplementary Information

Supplementary Information.

## Data Availability

The datasets used and/or analysed during the current study available from the corresponding author on reasonable request.
