# Supplementary material for: Noncontact assessment for fatigue based on heart rate variability using IR-UWB radar
Source: Sci Rep. 2022 Aug 20;12:14211. doi: 10.1038/s41598-022-18498-w (PMC9392064; doi:10.1038/s41598-022-18498-w)
Supplement: Supplementary file 1 — Supplementary Information. [file 41598_2022_18498_MOESM1_ESM.pdf]

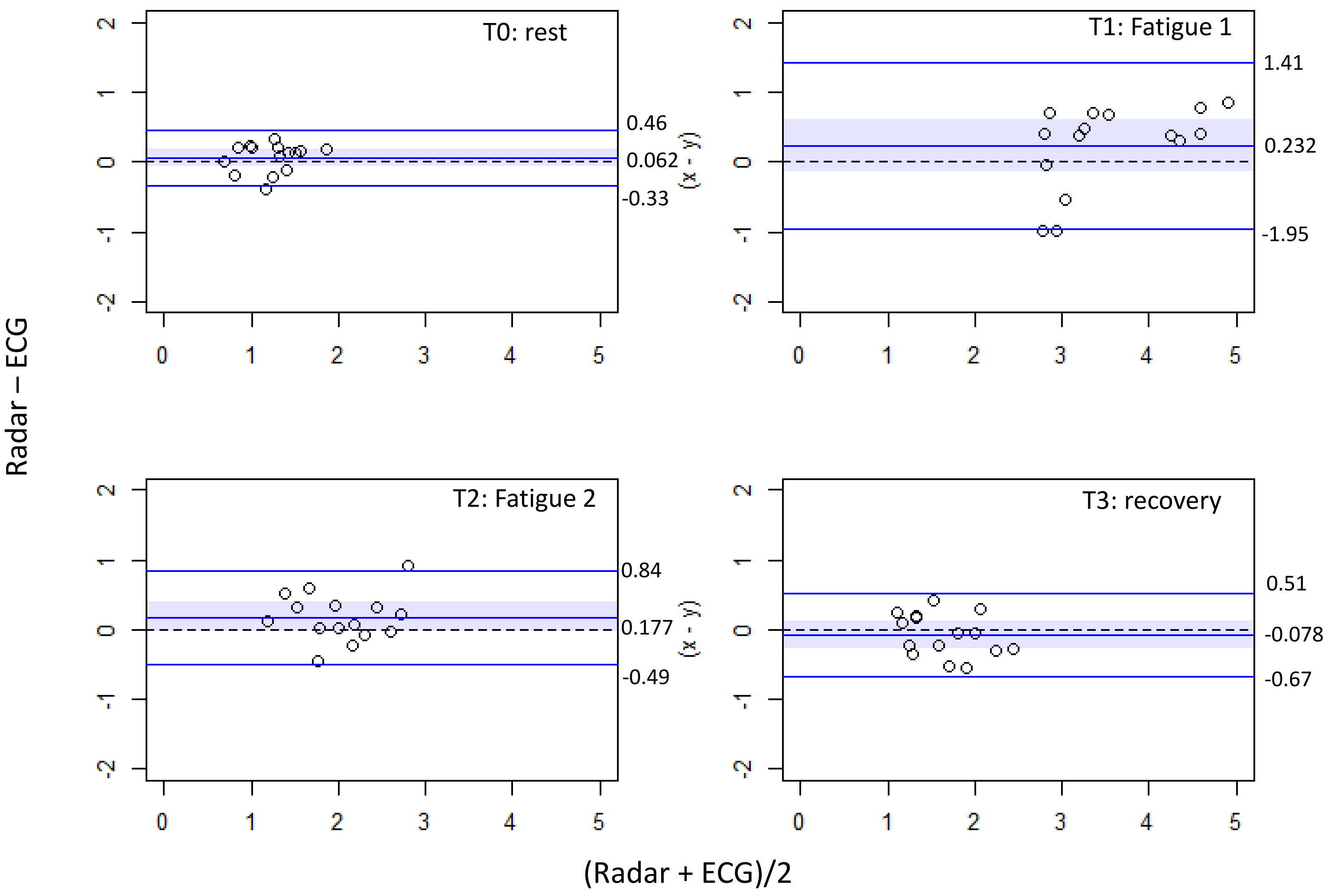

Supplementary Fig 1. Bland-Altman plots for agreement of HRV between radar and ECG

The blue solid lines indicate the mean bias and the upper and lower 95% LOA, the blue ribbons indicate the 95% confidence intervals of the mean bias.

There were no significant mean biases between radar and ECG.
